# Exploring e-sports fans’ motivation for watching live streams based on self-determination theory

**DOI:** 10.1038/s41598-024-64712-2

**Published:** 2024-06-15

**Authors:** Yuxin Tang, Xiaowen Zhang, Shengfeng Zan

**Affiliations:** 1 School of Physical Education and Sports Science, South China Normal University, Guangzhou, China; 2 School of International Studies, Renmin University of China, Beijing, China; 3 School of Physical Education, Shandong University, Jinan, China

**Keywords:** Self-determination theory, E-sports fans, Live streaming viewing, Intrinsic motivation, Extrinsic motivation, Psychology, Human behaviour

## Abstract

This study seeks to examine the multifaceted influences of diverse motivational factors on the live streaming engagement of e-sports fans based on self-determination theory. While previous research has focused on the offline participation in e-sports events, the shift towards live streaming engagement has created a new and underexplored area: the motivations for live streaming viewing among e-sports fans. Consequently, this research develops an e-sports Live Streaming Viewing Motivation Scale for evaluating both intrinsic and extrinsic motivations underlying e-sports fans’ live streaming engagement, and then utilises SPSS 26.0 and AMOS 26.0 to assess the reliability and validity of the scale. Subsequently, multiple linear regression analysis of 1052 questionnaires is employed to construct models and evaluate hypotheses. Findings indicate that : (1) Intrinsic motivation exhibits significant impact on the live viewing behaviour of e-sports fans. (2) However, the impact of extrinsic motivation is insignificant. (3) When both intrinsic and extrinsic motivations coexist, intrinsic motivation maintains a positive impact, whereas extrinsic motivation demonstrates a negative influence. (4) The motivational influence is multifaceted; notably, dimensions such as idol worship, leisure entertainment, and competitive stimulation positively affect live viewing motivation, while belonging identification, social engagement, and peripheral activities exert a negative impact. In conclusion, intrinsic motivation emerges as the primary driving force behind e-sports fans’ live streaming viewing behaviour. Extrinsic motivation fails to independently influence live streaming engagement and even dampens enthusiasm when combined with intrinsic motivation. Theoretically, this study contributes to the existing literature on Self-determination theory and motivations behind e-sports live streaming viewing behaviour. It not only refines the motivation scale, but also elucidates the impact of various motivations on viewing behaviour. Practically, it provides insights for optimising e-sports products and services.

## Introduction

In China, e-sports has emerged as a popular sport pursuit, capturing the interest of a vast majority of young people. In 2020, the Olympic Council of Asia officially recognised e-sports as a competitive discipline for the Asian Games. At the 19th Hangzhou Asian Games, China’s e-sports team clinched 4 gold medals and 1 bronze medal, bringing honor to the nation and catalyzing the growth of China's e-sports industry. The e-sports sector in China is experiencing rapid development, boasting a large number of e-sports enthusiast groups. According to the China e-sports Industry Report 2022, the number of e-sports users in China is projected to reach 488 million in 2022, with industry revenue expected to reach 144.503 billion yuan. As the frequency of e-sports events increases, more fans are drawn to spectate. However, geographical and time constraints often hinder offline attendance. Consequently, the popularity of e-sports webcasting has surged, with fans favoring devices such as mobile phones or computers to watch live events on online platforms such as Tiger Tooth Live, Penguin Gaming, and Hummingbird Gaming.

The motivation for sports events attendance serves as a fundamental driver, activating, guiding, and adjusting the behaviour of sports event audiences^1^. Initially, during the nascent stages of e-sports development, scholarly attention was predominantly directed towards understanding motivations for traditional sports events. Scholars delved into audience motivations across various traditional sports events, such as the Olympics, exploring facets like idol worship and cultural identity^2–4^. They also crafted several scales to gauge motivations for watching traditional sports events. Notably, the SPEED scale developed by Funk^2,3^ probed motivations spanning Socialisation, Performance, Excitement, Esteem and Diversion. Moreover, Sport Fan Motivation Scale (SFMS) developed by Wann^5^ includes broader dimensions including self-esteem, entertainment, belongingness, family, economics, eustress, diversion, and aesthetics.With the swift advancement of digital technology, scholarly focus has increasingly shifted towards intrinsic motivations underlying attendance at e-sports events. Drawing from the framework of traditional sports event motivations, researchers have investigated motives for attending e-sports events and developed validated scales for such motivations^6^. Grounded in satisfaction theory, seven motivations—such as knowledge acquisition, identification, and entertainment—were delineated for e-sports participants^7^. Further, Lee and Schoenstedt^8^ revealed fourteen motivations through a comparative analysis of motivations for participating in traditional sports events versus e-sports events.

Yet, as e-sports live streaming emerges as the primary means for fans to engage with the e-sport, motivations for watching e-sports live streams have surfaced as a novel research avenue necessitating further exploration and study. Despite the fact that scholars have currently conducted in-depth research on participation motivations in traditional sports events^9,10^ and fan’s motivations for watching traditional sports events^11^, there is a paucity of studies on the motivational factors driving e-sports live streaming viewing. Given the distinctions between watching e-sports live streams and traditional sports events, which encompass competition format, viewing channels, entertainment quotient, and experiential elements, motivations elucidated for traditional sports events might not entirely align with those pertinent to e-sports live streaming^6^.. Therefore, there exists an urgent need for in-depth research in the new area of motivations for e-sports live streaming watching behaviour. Such an endeavour has the potential to make a significant contribution to the existing literature, because it represents a pioneering and exploratory research initiatives, shedding light on an aspect of e-sports culture that remains relatively under-explored.

The objective of this study is thus to delve into the driving forces behind e-sports fans’ engagement with live streams, examining the influence of these motivations on their behaviour, and elucidating their underlying significance. More specifically, this study firstly introduces Self-Determination Theory (SDT) that underscores the role of self-determination processes in guiding human behaviour, in which individuals make free choices about their actions based on full awareness of their personal needs and environmental information^12^. Moreover, drawing insights from existing research findings, this study revises and builds an E-sports Live Streaming Viewing Motivation Scale to explore the multidimensional motivations for e-sports fans to watch live streams. Using SPSS 26.0 and AMOS 26.0, the research then validates the scale, explores the impacts of different motivations, and analyses deeper implications from the e-sports fans’ intrinsic and extrinsic perspectives.

This study aims to make several contributions. Firstly, by crafting a comprehensive scale of motivations tailored specifically for e-sports fans’ engagement with live streams, it seeks to provide a valuable resource for future research endeavours in this domain. Secondly, by exploring the impact of different motivations on e-sports fans’ live streaming viewing behaviour and elucidating the underlying rationales, this study endeavours to enhance our understanding of the dynamics at play in the e-sports audience landscape. Lastly, the identification of key motivation factors is anticipated to not only enable the optimisation of e-sports live streaming products, but also facilitate the creation of content that resonates more effectively with the needs and preferences of e-sports fans, thus enhancing their viewing experience.

## Literature review

### Self-determination theory

SDT is a theory of individual behavioural motivation proposed by American psychologists Richard M. Ryan and Edward L. Deci in the 1980s based on psychological needs theory, integration theory, cognitive theory and attribution theory^13^. SDT emphasises the active role of the self in the motivational process. It is a potentiality concerning experiential choices, where individuals make free choices about their actions based on a thorough understanding of personal needs and environmental information^12^. Meanwhile, SDT elucidates the process by which individuals engage in behaviours from a motivational perspective, recognising the interplay between internal and external motivations in shaping the behaviours^14^. It values individuals’ inherent desire to pursue psychological growth and well-being, explaining the essential elements necessary to achieve that pursuit^12^. When people’s psychological needs are satisfied, the internal motivation of individuals will be significantly strengthened, leading to enhanced work efficiency, heightened motivation, and a greater sense of well-being^15^.

The ideas put forward by SDT are considered to be the basic psychological needs driving individuals’ participatory behaviour and have garnered significant scholarly attention across various disciplines including psychology, sociology, and sports studies. In the realm of psychology, for instance, SDT not only plays a pivotal role in facilitating personality development, enhancing psychological functioning, and fostering growth processes, but also holds substantial significance in guiding psychological interventions and treatments^16–18^. Moreover, in the field of sociology, SDT offers insights into the dynamics of romantic relationships, elucidating the exchange of self-perspectives and the interdependence inherent in optimal romantic partnerships^19^. In addition, it also finds application in workplace relationships, aiding individuals in self-assessment of their leadership capabilities and future career development trajectories^20^. Last but not least, within the sports studies, SDT contributes to the understanding of the intrinsic and extrinsic motivations underpinning sports behaviours such as participation and consumption, thereby guiding strategies to effectively motivate individuals to engage in sports activities^18,21^.

**Figure 1:**
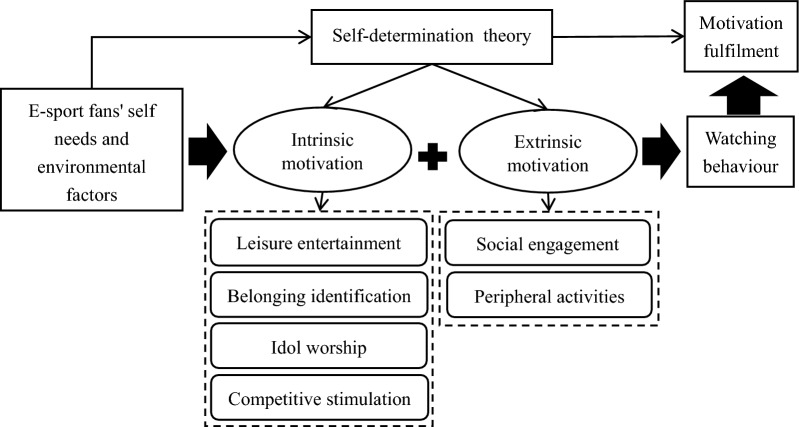
Theoretical framework of e-sports fans’ motivation to watch live streams.

In our case, SDT supports the argument that e-sports live streaming viewing is a behaviour voluntarily undertaken by individuals and influenced by various motivational factors. The decisions made by e-sports fans, following comprehensive consideration, resonate with the fundamental principles of SDT, which posits that behaviours are propelled by the fulfillment of personal needs. This theory provides a comprehensive framework encompassing intrinsic motivations, which involve satisfying inherent needs within the activity itself, and extrinsic motivations, which involve extrinsic factors influencing behaviour. This study thus constructs a theoretical framework for the motivations of e-sports fans to watch live streams based on SDT (see Fig. 1). The self-needs and environmental factors of e-sports fans give rise to intrinsic and extrinsic motivations, which subsequently drive the behaviour of watching e-sports events, ultimately fulfilling self-motivated needs. This theoretical framework, rooted in SDT, guides the entire process from the generation to the actual fulfilment of motivations in the live streaming viewing motivations of e-sports fans.

### Research hypotheses

#### Influence of intrinsic motivation

Intrinsic motivations are primarily explained as the pursuit of three needs: “value”, referring to self-benefits; “capability”, pertaining to task competence; and “pleasure”, related to enjoyable experiences^14^. Research has found that intrinsic motivations such as health, stress relief, emotional release, leisure entertainment, and sense of achievement among sports event audiences influence their enthusiasm for watching^22^. For example, the Sports Fan Motivation Scale (SFMS) developed by Wann^5^ explored the intrinsic motivations of sports event audiences from dimensions such as eustress, self-esteem, entertainment, aesthetics, escape from reality, and team belongingness. In the same vein, Trail and James^23^ suggested that intrinsic motivations such as learning, aesthetics, sense of belonging, and sense of achievement directly influence fans’ motivations for watching. Peng and Guo^24^ highlighted that the self-esteem motivation of e-sports fans directly affects their enthusiasm for watching. Liu et al.^25^ further added the factors of patriotism, entertainment, and learning into the motivational framework.

Based on the existing literature, the research analyses four dimensions of intrinsic motivation of e-sports fans’ live streaming viewing behaviour, namely, idol worship^23,26, 27^, leisure entertainment^2,3, 5, 7^, belonging identification^2,3, 6, 28^, and competitive stimulation^28–30^. Idol worship motivation refers to the identification and admiration that e-sports fans show towards professional e-sports athletes, virtual characters, and other subjects, demonstrated through their attention and emotional projection. Leisure entertainment motivation pertains to the desire of e-sports fans to fulfill their own needs, relieve stress, and relax both mentally and physically through watching live streams. Belonging identification motivation is associated with the process where e-sports fans perceive themselves as part of a certain e-sports community. Competitive stimulation motivation regards to the exhilarating feeling e-sports fans experience during live streams through exciting visuals and the competitive process.

The aforementioned studies collectively indicate that the intrinsic motivations of e-sports fans can directly and positively influence their enthusiasm for watching matches. In other words, the higher the intrinsic motivation of e-sports fans, the stronger their desire to watch live streaming matches and the more frequently they watch. This study thus posits Hypothesis 1:

##### H1

The intrinsic motivation of e-sports fans has a significant positive impact on their behaviour of watching live streams.

Considering the complexity of e-sports fans’ motivations for live streaming viewing, this study aims to better explore the specific intrinsic motivations behind the viewing behaviour of e-sports fans. Based on the analytical framework of e-sports fans’ viewing motivations proposed earlier, this study separately investigates the influences of idol worship, belonging identification, leisure entertainment, competitive stimulation. To this end, this paper disaggregates Hypothesis 1 into the following sub-hypotheses:

##### H1a

The motivation of idol worship among e-sports fans has a significant positive impact on live streaming viewing behaviour.

##### H1b

The motivation of leisure entertainment among e-sports fans has a significant positive impact on live streaming viewing behaviour.

##### H1c

The motivation of belonging identification among e-sports fans has a significant positive impact on live streaming viewing behaviour.

##### H1d

The motivation of competitive stimulation among e-sports fans has a significant positive impact on live streaming viewing behaviour.

#### Influence of extrinsic motivation

Extrinsic motivations encompass the drives that fulfill individuals’ external needs, such as socialisation, rewards, peripheral activities, and consumption^29–31^. Chen et al.^26^ demonstrated that external environmental factors of peripheral activities and consumption jointly influence the frequency of sports event viewing among fans. Additionally, factors like socialisation, aesthetics, media advertising, and behaviour of athletes have been identified as enhancing fans’ enthusiasm for watching matches^32^. Moreover, the sports atmosphere, sports event marketing activities, and community support have been shown to stimulate interest in watching matches and enhance enthusiasm among spectators^27,33^. Furthermore, Pu et al.^44^ found that external motivators, such as the atmosphere of e-sports events and social interaction, directly influence the enthusiasm of e-sports fans for watching.

In the same vain, the research summarises two dimensions of extrinsic motivation from the literature review, namely, social engagement^4,5,23^ and peripheral activities^6,29,30^. Social engagement motivation refers to the social behaviour of e-sports fans, such as sharing and interacting with family and friends about e-sports events. Peripheral activity motivation pertains to the participation of e-sports fans in peripheral activities during e-sports live streams, such as giveaways, lucky draw, and advertisements.

Since that the aforementioned studies jointly indicate extrinsic factors, whether environmental, social, and cultural, can exert a positive impact on the viewing motivations of e-sports fans, the article hypothesises that:

##### H2

The extrinsic motivation of e-sports fans has a significant positive impact on their behaviour of watching live streams.

Similarly, the H2 is divided into two sub-hypotheses that:

##### H2a

The motivation of peripheral activities among e-sports fans has a significant positive impact on live streaming viewing behaviour.

##### H2b

The motivation of social engagement among e-sports fans has a significant positive impact on live streaming viewing behaviour.

#### Combined influence of intrinsic and extrinsic motivation

The viewing motivations of e-sports fans can be multifaceted, often encompassing a combination of motivational factors. Previous studies have identified various complex motivational elements, such as socialisation, escapism, idolisation, and sense of achievement, as positive predictors of viewing behaviour among e-sports event audiences^29,30, 34^ analysed 13 “push and pull” factors among e-sports audience’ viewing behaviour, indicating a combination of both internal and external ones. Additionally, Koronios et al.^35^ found that multifaceted motivations, including attachment to a team, achievement, social factors, drama, and role models, influence audience’s consumption behaviour when watching sports events. Furthermore, Lu^36^ explored the relationship between viewing motivation and behavioural intention of spectators at e-sport events based on the SDT, arguing that both internal motivation and external perceived value of spectators had a significant positive effect on the behavioural intention to watch e-sport. Trail and James^23^ found that intrinsic motivations such as aesthetics, knowledge acquisition, achievement, and extrinsic motivations such as family, escapism, positive pressure, and social interaction directly influence viewing behaviour among sports event audiences. Collectively, above studies indicate that the viewing behaviour of e-sports fans is influenced by diverse motivational factors, encompassing both intrinsic motivations such as leisure entertainment, belonging identification, idol worship, competitive stimulation, and extrinsic motivations such as social engagement and peripheral activities. Based on the above research, this study proposes Hypothesis 3:

##### H3

The combined influence of intrinsic and extrinsic motivation has a significant positive impact on the behaviour of watching live streams among e-sports fans.

## Research methodology

### Questionnaire design

Drawing upon the review of existing literature and interviews with experts in sports science and e-sports fans, 28 question items from the six motivational dimensions have been decided to be included in the initial questionnaire designed for this study (see Table 1).

**Table 1:** E-sports live streaming viewing motivation scale.

Motivation dimension	Serial number	Topic content	Sources of questions
Idol worship	IW1	I have a favourite gaming player or team	Trail^23^, Tan et al.^27^, Chen et al.^26^
	IW2	Watching e-sport events to see my favourite e-sports players	
	IW3	Watching e-sport tournaments I want to see my favourite players perform well	
	IW4	I watch e-sport events to cheer on my favourite players	
	IW5	I'll pick a game with my favourite gamer in it	
	IW6	If my favourite e-sports player is not in the viewing arena it reduces my desire to watch the game	
Leisure entertainment	LE1	Pleasure and relaxation when watching e-sport events	Wann^5^, Funk et al.^2^,^3^, Kim^7^
	LE2	Getting thrills and excitement from watching e-sport events	
	LE3	Watching e-sport tournaments can regulate my mood and make me forget about life's troubles	
	LE4	Watching e-sport events is a form of entertainment	
	LE5	Watching e-sport tournaments online is a way for me to relieve stress at work	
Belonging identification	BI1	I want my country's team to win	Yang and Liu^6^, Funk et al.^2^,^3^, Xie^28^
	BI2	I'd rather see my favourite team win	
	BI3	I watch e-sport events where I can cheer on my favourite national teams and athletes	
	BI4	I'm very proud when my home team wins	
	BI5	I get frustrated when my home team loses	
Peripheral activities	PA1	The activities around e-sport events (celebrity singing, interactive fan games, raffles) are very appealing to me!	Qian et al^29,30^, Yang and Liu^6^
	PA2	Watching e-sport tournaments makes me want to buy peripheral products (dolls, clothes, etc.)	
	PA3	Activities during breaks in e-sport tournaments increase my motivation to watch the game	
	PA4	I'll watch the awards ceremony or post-game interviews at the end of an e-sport tournament	
Competitive stimulation	CS1	I love the uncertainty of winning and losing e-sport tournaments	Qian et al^29,30^, Xie^28^
	CS2	I enjoy watching e-sport tournaments that are at a comparable level of competitiveness and excitement	
	CS3	I'm hoping for a headwind or a turnaround	
	CS4	I love the thrill of the e-sport tournament process	
Social engagement	SE1	I like to watch e-sport events with friends and family	Wann^5^, Kahle^4^; Trail^23^
	SE2	I watch e-sport events because I watch everything around me	
	SE3	Watching e-sport tournaments would give my friends and I more things to talk about together	
	SE4	Watching e-sport events makes me more interested in talking about e-sports with others	

The questionnaire contains two parts. The first part solicits demographic characteristics such as gender, age, occupation, education level, monthly income, and preferred e-sports types, totaling six items. The second part consists of the E-sports Live Streaming Viewing Motivation Scale, drawing inspiration from previous studies by Trail^23^, Chen et al.^26^, Wann^2,3, 5, 7, 29, 30^, Yang and Liu^6^, Xie^28^, and Kahle^4^. The scale encompasses six dimensions: idol worship, leisure entertainment, belonging identification, competitive stimulation, peripheral activities, and social engagement, comprising a total of 28 items. The Likert 5-point scoring method was employed for the scale in the second part of the questionnaire, with scores ranging from 1 to 5 indicating “strongly disagree” to “strongly agree”, where higher scores denote higher viewing motivations. To ensure the reliability and validity of the questionnaire, feedback from relevant experts, e-sports event organizers, and e-sports fans was sought to assess the accuracy and scientific rigor of the questionnaire items. Based on the received feedback, necessary revisions were made to the questionnaire.

### Sample and data collection

This study targeted e-sports fans as its research subjects and utilised simple random sampling and snowball sampling methods to gather samples through both face-to-face and online questionnaire surveys. For the online questionnaire, a questionnaire link and QR code were generated using the Chinese questionnaire collection platform WJX to facilitate online surveys. The research process was divided into two stages: pilot research and formal research. During the preliminary research phase, a small-scale survey was conducted to assess the reliability and validity of the questionnaire, thereby ensuring its credibility. While the formal investigation is to explore the influence of different motivations on the live watching behavior of e-sports fans.

#### Pilot research

The pilot survey was conducted from July 14, 2023 to July 22, 2023, covering provinces such as Shandong, Sichuan, Henan, Jiangsu, Guizhou, and Guangdong, utilising a combination of offline and online methods. A total of 275 questionnaires were distributed during the preliminary research phase. After excluding invalid questionnaires due to incomplete responses or identical answers to all items, a total of 196 valid questionnaires were obtained. Among these, 152 respondents identified as male e-sports fans, while 44 identified as female e-sports fans. Adolescents and young adults accounted for 88.8% of the surveyed e-sports fans, whereas middle-aged and elderly individuals comprised 11.2%. Approximately 79.1% of the respondents were students. In terms of e-sports preferences, 80.6% of the fans enjoyed playing multiplayer online tactical competitive games, 59.2% favored first-person shooter games, 17.3% enjoyed real-time strategy games, and 18.4% preferred collectible card games.

#### Formal research

Since that e-sports events maily encompass four categories: multiplayer online tactical games, first-person shooter games, real-time strategy games, and collectible card games, this study conducted a formal questionnaire survey targeting each type of e-sports fans from August 3, 2023, to September 16, 2023. The formal survey distributed 1,399 questionnaires through random sampling across more than twenty provinces in China. After eliminating invalid questionnaires, such as those with missing information or identical responses for all items, a total of 1,052 valid questionnaires were collected, resulting in a validity rate of 75.2%.

The respondents’ demographic characteristics include gender, age, occupation, education level, monthly income, and preferred type of e-sports. Of the respondents, 59.7% were male and 40.3% were female, which was due to the gender ratio difference of e-sports fans observed in reality. The majority of respondents (82.7%) were aged below 35, reflecting the inclination of teenagers towards electronic gaming, which aligns with the current e-sports landscape in China. Among the respondents, 57.3% were students, 53.3% held a bachelor's degree, and approximately half belonged to the income group earning below 3,000 yuan. Undergraduate students typically experience less academic pressure and have more time for e-sports gaming, albeit with lower incomes. In terms of preferred e-sports games, 67.5% of respondents favored multiplayer online tactical games, followed by 56.6% for first-person shooter games, 32.9% for real-time strategy games, and 29.6% for collectible card games. Some e-sports fans may enjoy playing multiple types of e-sports games (see Table 2).

**Table 2:** Demographic characteristics of respondents.

Norm	Variant	Frequency	Percentages (%)	Cumulative percentage (%)	M	SD
Sex	Male	628	59.7	59.7	1.40	0.49
	Females	424	40.3	100.0		
Age	Under 18	134	12.7	12.7	2.48	1.19
	18–25 years	592	56.3	69.0		
	26–35 years	144	13.7	82.7		
	36–45 years	78	7.4	90.1		
	46–55 years	73	6.9	97.1		
	55 years and over	31	2.9	100.0		
Careers	National public officials	136	12.9	12.9	4.04	1.59
	Private sector employees	119	11.3	24.2		
	Profession	66	6.3	30.5		
	Self-employed person	55	5.2	35.7		
	Students	603	57.3	93.1		
	Else	73	6.9	100.0		
Education level	Junior high school and below	33	3.1	3.1	3.51	0.98
	High school or secondary school	169	16.1	19.2		
	Three-year college	182	17.3	36.5		
	Undergraduate	561	53.3	89.8		
	Master's degree or above	107	10.2	100.0		
Monthly salary	Below RMB 1500	399	37.9	37.9	2.49	1.45
	RMB 1500–3000 yuan	177	16.8	54.8		
	RMB 3,000–6,000 yuan	164	15.6	70.3		
	RMB 6000–10,000	184	17.5	87.8		
	RMB 10,000 or more	128	12.2	100.0		
Favourite game types	Multiplayer online tactical competitive game	710	67.5	67.5	0.67	0.47
	First person shooter	595	56.6	124.1	0.57	0.50
	Real-time strategy games	346	32.9	157.0	0.33	0.47
	Collectible card game	311	29.6	186.6	0.30	0.46

#### Data analysis

This study employed reliability analysis, exploratory factor analysis (EFA), and confirmatory factor analysis (CFA) using SPSS 26.0 and AMOS 26.0 to assess the reliability and validity of the questionnaire scales. Multiple linear regression analysis was then conducted using SPSS 26.0 to explore the influence of intrinsic motivation and extrinsic motivation, respectively, and the co-influence of intrinsic and extrinsic motivation of e-sports fans on the frequency of watching e-sports live streaming events. Moreover, the study also examined impacts of the six motivational dimensions separately, including leisure entertainment, belonging identification, social engagement, idol worship, peripheral activities, and competitive stimulation. A model was constructed to test the hypotheses.

## Results

### Descriptive statistics

In this study, descriptive statistics were performed on the core variables (see Table 3), including the mean, standard deviation, skewness, and kurtosis of each variable. The mean value of the respondents’ questions on the scale was above 3.29, indicating a high level of agreement on the motivation of e-sport fans for live viewing. Kline^37^ showed that when the absolute value of the skewness of the data is less than 3 and the absolute value of the kurtosis is less than 10, the sample is considered to essentially satisfy a normal distribution. From the descriptive statistics of the core variables of the data, it can be found that the absolute value of the skewness of the e-sport tournament viewing motivation scale is 1.114 at the maximum, and the absolute value of the kurtosis is 1.203 at the maximum, and the absolute value of the skewness and kurtosis of all the question items of the scale are in line with the standard range, and the data are approximated to be normally distributed, which allows for the corresponding statistical analyses of the data.

**Table 3:** Descriptive statistics of core variables.

Motivation dimension	Serial Number	Average value	Standard deviation	Skewness		Kurtosis	
				Statisticians	Standard error	Statisticians	Standard error
Idol worship	IW1	3.580	1.026	− 0.433	0.075	− 0.306	0.151
	IW2	3.590	1.043	− 0.478	0.075	− 0.259	0.151
	IW3	3.700	0.994	− 0.611	0.075	0.104	0.151
	IW4	3.520	1.018	− 0.339	0.075	− 0.273	0.151
	IW5	3.640	0.975	− 0.583	0.075	0.093	0.151
	IW6	3.570	1.019	− 0.451	0.075	− 0.159	0.151
Leisure entertainment	LE1	3.800	0.881	− 0.668	0.075	0.553	0.151
	LE2	3.670	0.974	− 0.562	0.075	0.042	0.151
	LE3	3.640	0.933	− 0.406	0.075	− 0.116	0.151
	LE4	3.810	0.895	− 0.697	0.075	0.462	0.151
	LE5	3.680	0.927	− 0.455	0.075	− 0.024	0.151
Belonging identification	BI1	4.150	0.928	− 1.114	0.075	1.033	0.151
	BI2	3.980	0.982	− 0.857	0.075	0.240	0.151
	BI3	4.010	0.959	− 0.912	0.075	0.535	0.151
	BI4	4.000	0.943	− 0.849	0.075	0.455	0.151
	BI5	3.730	0.991	− 0.502	0.075	− 0.237	0.151
Peripheral activities	PA1	3.390	1.066	− 0.360	0.075	− 0.386	0.151
	PA2	3.290	1.126	− 0.183	0.075	− 0.671	0.151
	PA3	3.370	1.064	− 0.215	0.075	− 0.502	0.151
	PA4	3.460	1.068	− 0.395	0.075	− 0.376	0.151
Competitive stimulation	CS1	3.820	0.949	− 0.757	0.075	0.434	0.151
	CS2	3.830	0.950	− 0.693	0.075	0.316	0.151
	CS3	3.900	0.935	− 0.715	0.075	0.282	0.151
	CS4	3.860	0.905	− 0.691	0.075	0.510	0.151
Social engagement	SE1	3.730	0.960	− 0.681	0.075	0.332	0.151
	SE2	3.460	1.068	− 0.365	0.075	− 0.437	0.151
	SE3	3.620	0.966	− 0.526	0.075	0.040	0.151
	SE4	3.660	0.946	− 0.541	0.075	0.049	0.151

### Reliability and validity tests

#### Reliability analysis

Reliability analyses were conducted in this study using SPSS 26.0. Firstly, the internal consistency Cronbach's α coefficient was used to test the questionnaire, yielding a value of 0.955 for the questionnaire as a whole, with Cronbach's α coefficients for the six factors exceeding 0.8. Secondly, the Corrected Item-Total Correlation (CITC) values of the analysed items were all greater than 0.4, indicating a strong correlation between the analysed items. Finally, when examining the Cronbach’s α coefficient after deletion of items, no significant increase in the reliability coefficient was observed after deleting any question item. In conclusion, the internal consistency of the questionnaire in this study is deemed relatively good.

#### Validity analysis

- (1) Content validity

The items on the questionnaire scale were designed based on established scales from previous research and were validated and revised through discussions with experts in the relevant field. Therefore, it can be considered to have good content validity.

- (2) Exploratory factor analysis

In this study, SPSS 26.0 was used to conduct EFA on 196 data from the preliminary study. The suitability of each variable for exploratory factor analysis was determined based on the KMO value and Bartlett’s sphere test. The results showed that the KMO value of 0.923 and Bartlett's sphere test approximate chi-square value of 4609.359 with a degree of freedom of 378 and a significance value of P=0.000<0.05, meeting the criterion of significance. These results indicate that the dataset is suitable for EFA.

The principal component analysis of the 28 question items conducted via EFA was rotated, revealing six factors with initial eigenvalues greater than or equal to one. The cumulative explained variance accounted for by these six factors was 76.703%, exceeding the 60% criterion. Therefore, the above results indicate that the six factors extracted from the 28 question items are more satisfactory for the interpretation of the raw data and fit with the six dimensions proposed in this study (see Table 4).

**Table 4:** Factor rotation for exploratory factor analysis.

Factors	Initial eigenvalue			Extract the sum of the squares of the loads			Rotational load sum of squares		
	Total	Percentage of variance	Cumulative percentage (%)	Total	Percentage of variance	Cumulative percentage (%)	Total	Percentage of variance	Cumulative percentage (%)
1	12.898	46.064	46.064	12.898	46.064	46.064	4.392	15.684	15.684
2	3.233	11.547	57.611	3.233	11.547	57.611	3.820	13.643	29.328
3	1.748	6.241	63.852	1.748	6.241	63.852	3.816	13.629	42.957
4	1.386	4.950	68.802	1.386	4.950	68.802	3.596	12.844	55.801
5	1.212	4.329	73.131	1.212	4.329	73.131	3.463	12.366	68.167
6	1.000	3.572	76.703	1.000	3.572	76.703	2.390	8.536	76.703
Extraction method: Principal component analysis									

The factor attribution of the preliminary study scale can be determined based on the rotated component matrix. The variables were rotated orthogonally using Kaiser’s normalised maximum variance method, with each factor loading above 0.5 after rotation. Factor 3 consisted of 5 variables with factor loadings ranging from 0.727 to 0.822 and was named “leisure entertainment”; Factor 4 consisted of 4 variables with factor loadings ranging from 0.747 to 0.831 and was named “peripheral activities”. Factor 5 consisted of 4 variables with factor loadings ranging from 0.740 to 0.804 and was named “competitive stimulation”; Factor 6 consisted of 4 variables with factor loadings ranging from 0.584 to 0.688 and was named “social engagement”. In summary, the questionnaire scale was categorised into six factors, with a clear dimensional structure that is basically consistent with the expectation of the scale dimension (see Table 5).

- (3) Confirmatory factor analysis

**Table 5:** Factor loadings for exploratory factor analysis.

Factor	Entry	Ingredient					
		1	2	3	4	5	6
Idol worship	IW1	0.740					
	IW2	0.795					
	IW3	0.778					
	IW4	0.709					
	IW5	0.752					
	IW6	0.709					
Leisure entertainment	LE1		0.770				
	LE2		0.772				
	LE3		0.747				
	LE4		0.732				
	LE5		0.726				
Belonging identification	BI1			0.769			
	BI2			0.727			
	BI3			0.822			
	BI4			0.773			
	BI5			0.751			
Peripheral activities	PA1				0.828		
	PA2				0.831		
	PA3				0.827		
	PA4				0.747		
Competitive stimulation	CS1					0.740	
	CS2					0.799	
	CS3					0.803	
	CS4					0.804	
Social engagement	SE1						0.584
	SE2						0.688
	SE3						0.650
	SE4						0.683

**Figure 2:**
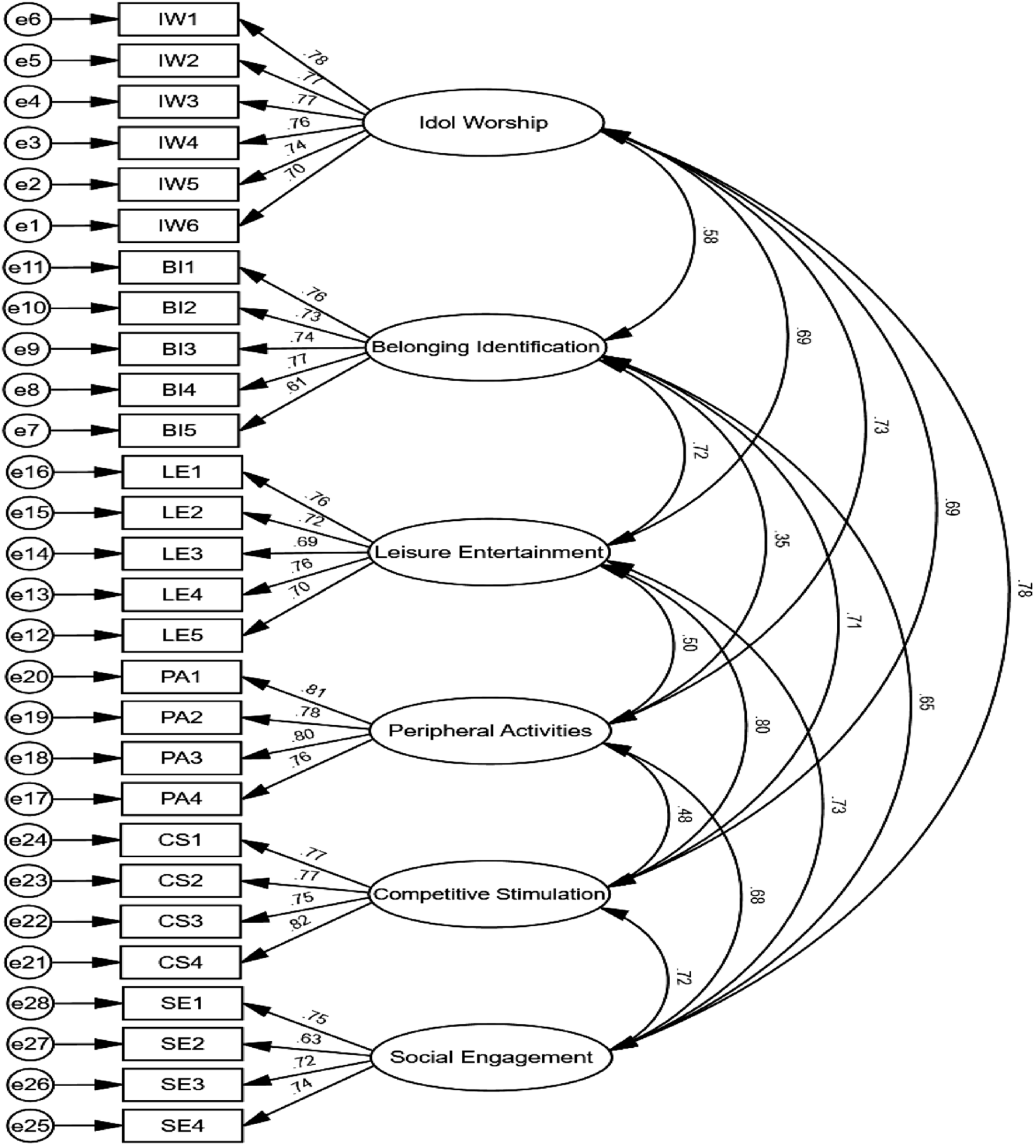
Standardised model of the first-order confirmatory factor.

**Figure 3:**
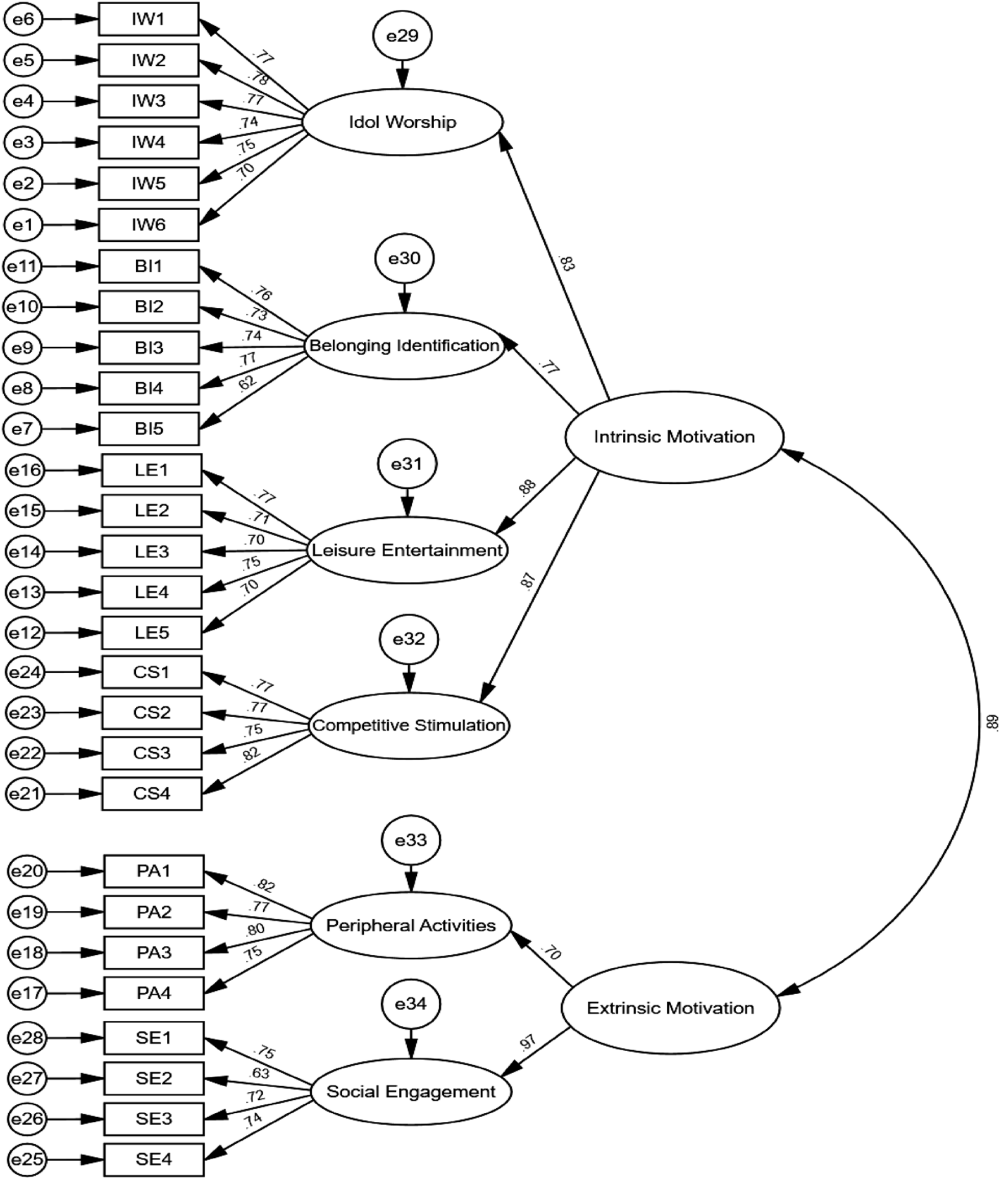
Standardised model of the second-order confirmatory factor.

In this study, AMOS 26.0 was employed to conduct CFA of the formal questionnaire data. The model was considered to have a good fit when the sample size in the CFA was greater than 200, X2/df was less than 5, RMSEA was less than 0.080, and NFI, RFI, CFI, IFI, and TLI were greater than 0.900^38^. The results showed that the results of the fit index of the first-order model were X2/df = 2.780, RMSEA = 0.041, NFI = 0.943, RFI = 0.935, CFI = 0.962, IFI = 0.963, and TLI = 0.958, suggesting that the model constructed in this study has a good degree of fitness (see Fig. 2). In addition, this study constructed a second-order factor model based on the first-order factor model (see Fig. 3), and the second-order model of e-sport viewing motivation was well adapted (see Table 6). In conclusion, the structural validity of the E-sport Live Viewing Motivation Scale is considered satisfactory.

**Table 6:** Confirmatory factor analysis coefficients.

	X2/df	RMSEA	NFI	RFI	CFI	IFI	TLI
Standard value	< 5.000	< 0.080	> 0.900	> 0.900	> 0.900	> 0.900	> 0.900
First-order model	2.780	0.041	0.943	0.935	0.962	0.963	0.958
Second-order model	3.433	0.048	0.928	0.920	0.947	0.948	0.942

Through the CFA, the factor loadings of the E-sports Live Streaming Viewing Motivation Scale were obtained. The factor loadings corresponding to the question items of each latent variable in the dimensions of idol worship, belonging identification, leisure entertainment, competitive simulation, peripheral activities, and social engagement were all higher than 0.6, indicating that the belonging topics corresponding to their variables are highly representative of their respective dimensions. In addition, the average variance extracted (AVE) for each variable extended 0.5 and the combined reliability CR was greater than 0.8. The results suggest that the convergent validity of the E-sport Live Streaming Viewing Motivation Scale is robost and desirable (see Table 7).

**Table 7:** Factor loadings for the E-sports live streaming viewing motivation scale.

Trails			Estimate	AVE	CR
F1	< --	F7	0.831	0.702	0.904
F2	< --	F7	0.771		
F3	< --	F7	0.878		
F5	< --	F7	0.868		
F4	< --	F8	0.703	0.720	0.833
F6	< --	F8	0.972		
IW6	< --	F1	0.700	0.566	0.886
IW5	< --	F1	0.748		
IW4	< --	F1	0.740		
IW3	< --	F1	0.771		
IW2	< --	F1	0.780		
IW1	< --	F1	0.769		
BI5	< --	F2	0.615	0.526	0.846
BI4	< --	F2	0.770		
BI3	< --	F2	0.741		
BI2	< --	F2	0.729		
BI1	< --	F2	0.760		
LE5	< --	F3	0.699	0.527	0.847
LE4	< --	F3	0.754		
LE3	< --	F3	0.696		
LE2	< --	F3	0.710		
LE1	< --	F3	0.766		
PA4	< --	F4	0.751	0.617	0.866
PA3	< --	F4	0.796		
PA2	< --	F4	0.775		
PA1	< --	F4	0.818		
CS4	< --	F5	0.824	0.606	0.860
CS3	< --	F5	0.752		
CS2	< --	F5	0.769		
CS1	< --	F5	0.769		
SE4	< --	F6	0.739	0.508	0.804
SE3	< --	F6	0.725		
SE2	< --	F6	0.633		
SE1	< --	F6	0.747		

As depicted in Table 8, there is a significant correlation between idol worship, belonging identification, leisure entertainment, competitive stimulation, peripheral activities, and social engagement (*p* < 0.01). In addition, the absolute values of the correlation coefficients for each variable are less than 0.5 and are all lower than the square root of the corresponding AVEs. This indicates a certain degree of correlation among the variables while also demonstrating sufficient differentiation between them. In conclusion, the e-sports live viewing motivation scale exhibits good discriminant validity.

**Table 8:** Correlation and discriminant validity test for E-sport live streaming viewing motivation scale (N = 1052).

	F1	F2	F3	F4	F5	F6
F1	0.566					
F2	0.252**	0.526				
F3	0.321**	0.281**	0.527			
F4	0.425**	0.170**	0.260**	0.617		
F5	0.368**	0.319**	0.382**	0.289**	0.606	
F6	0.394**	0.275**	0.329**	0.387**	0.377**	0.508
M	3.600	3.974	3.719	3.377	3.850	3.617
SD	0.808	0.753	0.726	0.912	0.784	0.780
AVE square root	0.752	0.725	0.725	0.785	0.778	0.713

**represents *p* < 0.01; Diagonal line is AVE evaluation variance variance extractions.

### Influence of e-sport fans’ motivation for live streaming viewing

This study examines the frequency of watching e-sports live events among e-sports fans as the dependent variable, while controlling for variables such as gender, age, occupation, education, and monthly income of e-sports fans. Different independent variables were separately incorporated into the model for multiple linear regression analysis. Four models are constructed as follows. Model 1: the independent variable is intrinsic motivation; Model 2: the independent variable is extrinsic motivation; Model 3: the independent variables are intrinsic motivation and extrinsic motivation; Model 4: the independent variables are the six dimensions of the motivations. Through regression analysis of the four models, the study investigates the variations in the influence of intrinsic motivation, extrinsic motivation, and each dimension of the motivations on the frequency of watching e-sports events among e-sports fans. The regression results are presented in Table 9 for detailed examination.

1. Intrinsic motivation showed a significant positive effect (β = 0.393, *P* < 0.01) on the frequency of watching live events (Model 1). This indicates that stronger intrinsic motivation correlates with an increased number of times e-sports fans engage with live events. H1 is thus supported.
2. Extrinsic motivation was found to be insignificant (β = 0.086, *P* > 0.1) in affecting the frenquency of watching live events (Model 2), suggesting that extrinsic motivation does not influence how often e-sport fans watch live events. H2 is not supported.
3. As indicated by Model 3, intrinsic motivations maintained a significant positive effect on the frequency of watching live streaming e-sports events (β = 0.652, *P* < 0.01). In contrast, extrinsic ones showed a significant negative effect (β = -0.318, *P* < 0.01). Compared to Model 1, the intrinsic motication in Model 3 exerted stronger positive effect on live streaming viewing behaviour of e-sports fans. Compared to Model 2, the impact of extrinsic motivation became significant but negative. The results underscore that when intrinsic motivation and extrinsic motivation co-influence the live streaming viewing behaviour, stronger intrinsic motivation leads to more frequent viewing behaviour. Conversely, stronger extrinsic motivation reduces the frequency of live streaming viewing behaviour. H3 is not supported.
4. To further validate the main dimensions of intrinsic motivation and extrinsic motivation that influence the frequency of e-sport fans watching e-sport live streaming events, the six dimensions were used as independent variables to construct regression model 4. The results showed that the idol worship (β = 0.456, *P* < 0.01), leisure entertainment (β = 0.255, *P* < 0.01), and competitive stimulation (β = 0.269, *P* < 0.01) of intrinsic motivation showed a significant positive effect on the number of e-sport live streaming viewings under the control of the viewer’s basic situation, supporting H1a, H1b, H1d. However, the intrinsic motivation, belonging identification, exhibited a significant negative effect on the frequency of e-sport live stream viewing (β = − 0.260, *P* < 0.01). H1c was denied. Moreover, the extrinsic motivations, including peripheral activity (β = -0.230, *P* < 0.01), and social engagement (β = − 0.215, *P* < 0.01) also showed significant negative impacts, contradicting H2a and H2b These findings underscore that intrinsic motivation dimensions such as idol worship, leisure entertainment, and competitive stimulation are the main drivers for e-sports fans to watch live streaming events. Conversely, the extrinsic motivation dimensions of peripheral activities and social engagement tend to reduce the motivation for e-sports fans to watch these events.

**Table 9:** Regression analyses of e-sport fans’ motivation and frequency to watch live streams.

Implicit variable	Number of times you watched live gaming events			
	Model 1	Model 2	Model 3	Model 4
Sex	− 0.539***	− 0.535***	− 0.532***	− 0.506***
Age	− 0.071	− 0.084*	− 0.077*	− 0.090**
Careers	− 0.110***	− 0.117***	− 0.121***	− 0.120***
Education level	0.026	0.03	0.012	0.018
Monthly salary	0.018	0.024	0.034	0.033
Multiplayer online tactical competitive game	0.405***	0.437***	0.412***	0.406***
First person shooter	0.732***	0.730***	0.772***	0.713***
Real-time strategy games	0.385***	0.389***	0.397***	0.388***
Collectible card game	0.414***	0.487***	0.398***	0.396***
Internal motive	0.393***		0.652***	
External motivation		0.086	− 0.318***	
Idol worship				0.456***
Leisure entertainment				− 0.260***
Belonging identification				0.255***
Peripheral activities				− 0.230***
Competitive stimulation				0.269***
Social engagement				− 0.215***
n	1052	1052	1052	1052
R^2^	0.197	0.172	0.208	0.235
AdjR^2^	0.189	0.164	0.199	0.224
F	25.464	21.674	24.765	21.182

## Conclusions and discussion

### Conclusion

This study, rooted in SDT, delves into the motivational factors influencing e-sports fans’ behaviour of watching live e-sports events. It begins by developing an e-sports live viewing motivation scale based on existing research. Subsequently, theoretical hypotheses are proposed by integrating intrinsic motivations (leisure entertainment, belonging identification, idol worship, competitive stimulation) and extrinsic motivations (social engagement, peripheral activities). These hypotheses are validated using survey data from 1052 e-sports fans through multiple linear regression modeling. The following conclusions emerge:

1. Intrinsic Motivation: Intrinsic motivation significantly and positively impacts e-sports fans’ behaviour of watching live streams. When intrinsic and extrinsic motivations coexist (Model 3), the influence of intrinsic motivation becomes even more pronounced. Therefore, the intrinsic motivation of e-sports fans emerges as the primary driving force behind their live viewing behaviour.
2. Extrinsic Motivation: Extrinsic motivation does not exert a significant influence on e-sports fans’ behaviour of watching live streams. However, when both intrinsic and extrinsic motivations are considered, extrinsic motivation demonstrates a significant negative impact. This suggests that extrinsic environmental factors may diminish e-sports fans’ enthusiasm for watching live e-sports matches.
3. Different Motivational Dimensions: Intrinsic motivation dimensions such as leisure entertainment, idol worship, and competitive stimulation positively affect e-sports fans’ behaviour of watching live broadcasts. Conversely, dimensions like belonging identification, social engagement, and peripheral activities exhibit a negative effect. From the perspective of SDT, e-sports fans tend to make choices based on their genuine desires, while extrinsic motivational factors may impede their live viewing behaviour. By uncovering the motivations behind e-sports fans, this study offers valuable insights for understanding and meeting the needs of this burgeoning community within the e-sports realm.

### Discussion

#### Intrinsic motivational influences on live streaming viewing by E-sports fans

The study’s findings regarding the significant positive effect of intrinsic motivation among e-sports fans on the frequency of live streaming viewings align with existing research^9,10,29,30,40^, particularly within the framework of SDT as explored by Lafrenière et al.^39^. With the proliferation of live streaming platforms, e-sports live streams have become increasingly accessible, prompting fans to engage with them more frequently. In this study, the intrinsic motivations of e-sports fans for live streaming viewing are categorised into four dimensions: idol worship, belonging identification, leisure entertainment, and competitive stimulation. These intrinsic motivations have shown significant positive effects on live streaming viewing behaviour, as they are closely tied to their personal interests, self needs, and satisfaction. For instance, with a profound interest in e-sports gaming and competition, e-sports fans are inherently drawn to watch live streaming events as they derive enjoyment and excitement from witnessing the games unfold. The results argue that intrinsic motivation stands out as the primary driver contributing to e-sports ethusiates’ engagement with live streaming. In this context, it becomes more imperative to cultivate sound values, provide correct insights into athletes’ competitive sportsmanship, and enhance the self-awareness through e-sports live streaming. These endeavors are anticipated to effectively promote e-sports events among the wider population.

#### Extrinsic motivational influences on live streaming viewing by e-sport fans

The findings reveal that the extrinsic motivation of e-sports fans for live streaming viewing has no significant effect on the frequency of e-sports live streaming viewing. Furthermore, it indicates that e-sports fans’ live streaming viewing is not influenced by social engagement and peripheral activities related to event viewing. This outcome contradicts the conclusions of most scholars who have posited that e-sports viewing is indeed influenced by extrinsic motivations,^9,10,31,40,41^. When considering only extrinsic motivation, e-sports fans do not actively engage in watching events via live streams. This can be attributed to the fact that e-sports fans possess a clear self-concept and follow their own stream of consciousness experience when wholeheartedly watching live e-sports events, as suggested by Lee et al.^42^. Additionally, e-sports fans can demonstrate strong autonomy in choosing to watch e-sports events on live streaming platforms, driven by their own interests and passions rather than external pressures or rewards. This further validates the argument that watching e-sports live streaming events is regarded as a recreational activity rather than a task-oriented one. Consequently, e-sports fans are unlikely to be coerced by external obligations to watch these live tournaments. Furthermore, with a wide diversity of features and the ubiquitous presence of the Internet, e-sports fans can conveniently access replays and edited highlights of tournaments at any location and at any time, free from the constraints of physical space or time. This enhanced flexibility significantly reduces the extrinsic motivation for e-sports fans to prioritise watching live events.

#### The combined influence of intrinsic and extrinsic motivations of E-sport fans for live streaming spectating

When intrinsic and extrinsic motivation are both investigated, intrinsic motivation exhibits a significant positive effect, while extrinsic motivation shows a significant negative effect. The findings of this study suggest that an increase in internal motivation among e-sports fans enhances their engagement with live e-sports events, whereas heightened extrinsic motivation diminishes the desire to watch e-sports. According to the analysis of SDT, the drive of e-sports fans to watch live events stems primarily from intrinsic motivation, which is associated with self-determination and personal growth. Whereas extrinsic factors may reduce the sense of satisfaction and achievement, subsequently dampening the motivation to watch events^43^. More specifically, the process of live viewing by e-sports fans often involves a deluge of extrinsic information and interaction, such as comments, discussions, and match statistics. This overflow of information can overwhelm viewers, leading to fatigue and impatience. Additionally, while external motives may enhance the engagement and social experience of e-sports fans, they can sometimes act as distractions and burdens on spectators watching the live streams^44^. Consequently, viewers need to strike a balance between social participation and the enjoyment derived from watching the matches.

#### Multi-dimensional influences on live streaming viewing by e-sport fans

The results of the study show that the idol worship, leisure entertainment, and competitive stimulation dimensions of intrinsic motivation have a significant positive effect on e-sport fans’ motivation for live streaming viewing; whereas, the belonging identification dimension of intrinsic motivation, and peripheral activities dimensions and social engagement dimension of extrinsic motivation have a significant negative effect on e-sport fans’ motivation for live streaming viewing. We discuss each of the six dimensions to explore the intrinsic reasons for their influence on e-sport fans’ motivation for live viewing.

1. Idol worship dimension: idol worship can stimulate the passion and motivation inherent in e-sport fans, who will be inspired by the stories, skills, and competitive spirit of the athletes. They are more inclined to emotionally invest in and empathise with the idol’s victories or challenges, thus increasing the motivation of e-sport fans to watch the live game^23^. In addition, since that e-sports fans may have a fondness for the appearance and skills of virtual game characters, they will feel a sense of self-affirmation or identification when watching live streams and seeing characters they excel at controlling and playing^45^.
2. Leisure entertainment dimension: first and foremost, e-sports fans watch live events for fun and relaxation, seeking an enjoyable experience to alleviate the pressures of their daily lives^41^. Secondly, live streaming provides convenience, allowing them to watch events anytime and anywhere without being restricted by time constraints, which further increases their motivation to watch. Finally, with the continuous advancement of VR virtual viewing and 4 K high-definition viewing technologies, e-sports fans can enjoy an immersive virtual game scene experience during live viewing, enhancing the overall e-sports viewing experience^46^.
3. Competitive stimulation dimension: as a competitive sporting event, e-sports captivates audiences who crave the high-level performances, intense competition, professional players’ exceptional skills, gameplay in actions, unexpected comebacks, and final outcomes^47^, which creates memorable moments through the live streaming watching. Additionally, some e-sports events feature exclusive skins and stunning special effects, enhancing the visual appeal and media coverage, thereby enriching the viewing experience and the overall competitive atmosphere^48^.
4. Belonging identification dimension: e-sports fans may experience emotional fluctuations when their supported teams or players perform poorly or lose the game. Therefore, the competitive pressure can dampen their desire to watch the live streams. Additionally, fans often feel a sense of responsibility to cheer for their supported teams or players, which can consume their time and energy, further diminishing their motivation to watch matches. Audience affiliation and identity are complex and multifaceted, requiring analysis across different contexts, locations, and events^49^.
5. Peripheral activity dimension: e-sports events are often subject to certain commercial activities, leveraging the influence of live streaming for product marketing and advertising to increase event revenue^50^. During e-sports event live streams, activities such as giveaways, product promotions, advertisements, and audience interactions may divert the attention of e-sports fans, causing them to miss crucial moments of the game. Consequently, this distraction can lead to viewer dissatisfaction and result in them tuning out of the match.
6. Social engagement dimension: e-sports events differ from traditional sports in terms of participation format, competitive effects, and event presentation, creating a gap between the virtual and the real world. Unlike the jubilant gatherings seen during events like the FIFA World Cup, e-sports fans prefer solitary experiences while watching live streams, reducing social engagement. This preference stems from the intrinsic nature of e-sports events^51^. In the context of e-sports live streaming, fans may interact with other viewers through chat rooms or social media, where negative comments or social behaviours can affect their emotions or distract their attentions. Furthermore, since most e-sports fans are adolescents, there is a generational gap in China where parents or elders may not approve of adolescents watching e-sports events, leading to conflicts and attempts to prevent them from continuing to watch^52^.

### Implications

This study offers both theoretical and practical implications. Theoretically, the study refined and validated the previously established e-sports enthusiast live viewing motivation scale, which possesses six dimensions and 28 items from the perspectives of both intrinsic and extrinsic motivation. In the meantime, succeeding in rigor validity and reliability tests, the scale provides a robust tool for future inquiries in this field. In addition, the research promoted the theoretical compatibility between SDT and motivations for e-sports live streaming viewing by constructing and testing models based on various motivational factors. Moreover, by clarifying the fundamental connotation of different motivations, the study enriches the theoretical framework of e-sports live streaming watching motivations. Practically, since the research findings highlight the key motivational factors driving e-sports fans, e-sports organisations can be empowered to tailor their offerings more effectively to meet the diverse needs of their audience, which will contribute to enhancing the live viewing experience and overall satisfaction of e-sports fans.

### Limitations and future research

As with any research endeavor, this study has several limitations that could be noticed. Firstly, while the six motivational factors among e-sports fans are identified relying on existing literature, they may potentially overlook other, more specific, and individual motivations and reasons for live streaming viewing. Viewing motivations may vary considerably among e-sports live streaming audiences based on their level of affection for e-sports, for instance, between dedicated loyal fans and casual viewers. Future research could address these limitations by incorporating a broader range of motivational variables to gain a comprehensive understanding of e-sports fans’ live viewing motivations. Additionally, further exploration could involve clearer categorisation of e-sports fans to investigate the viewing motivations of different segments more effectively.

### Ethics approval

The research is conducted in compliance with local laws, regulations, and institutional requirements, and it has received approval from the Ethics Committee of the university (Approval No: SCNU-SPT-2023-170).

### Informed consent

The individuals taking part in this research manifested their informed permission.

## Data Availability

The datasets used and analysed during the current study are included in the supplementary information, further inquiries can be directed to the corresponding author.
